# A Novel Hypothermic Preservation Formulation Containing SUL-138 Enables Long-Term Hypothermic Storage of Clinical-Grade CAR-T Cells

**DOI:** 10.3390/pharmaceutics18040414

**Published:** 2026-03-28

**Authors:** Aysenur Öner, Nina Nooteboom, Linette Oosting, Jos G. W. Kosterink, Bart G. J. Dekkers, Adrianus C. van der Graaf, Tom van Meerten, Guido Krenning, Daniel H. Swart, Robin Dennebos, Harm-Jan Lourens, Edwin Bremer, Bahez Gareb

**Affiliations:** 1 Department of Hematology, University Medical Center Groningen (UMCG), Hanzeplein 1 (DA13), 9713 GZ Groningen, The Netherlands; 2 Department of Clinical Pharmacy and Pharmacology, University Medical Center Groningen (UMCG), Hanzeplein 1 (AP50), 9713 GZ Groningen, The Netherlands; a.nooteboom@lumc.nl (N.N.); b.g.j.dekkers@umcg.nl (B.G.J.D.);; 3 Sulfateq B.V., Admiraal de Ruyterlaan 5, 9726 GN Groningen, The Netherlands

**Keywords:** hypothermic preservation, SUL-138, fresh CAR-T cells, stability, formulation buffer

## Abstract

**Background/Objectives:** Point-of-care (PoC) manufactured fresh chimeric antigen receptor (CAR)-T cells are typically formulated in hypothermic preservation formulations (HPFs) and stored under hypothermic conditions (2–8 °C) until administered to the patient. However, in current HPFs the shelf life of fresh CAR-T cells is short (~24–36 h) due to limited CAR-T cell stability, which poses significant time constraints on manufacturing procedures and logistics. The objective of this study was to improve the stability and extend the shelf life of fresh clinical-grade CAR-T cell drug products (DPs). **Methods:** A novel HPF was developed by supplementing a base HPF with the novel excipient SUL-138, which stabilizes mitochondria during hypothermic storage and subsequent rewarming, alone or in combination with endogenous mitochondrial substrates. This panel of HPFs was first screened for their stability-improving characteristics in the model cell line Jurkat cells. Subsequently, HPFs were assessed for their stability-improving characteristics of clinical-grade CD19 CAR-T cell DPs. Critical quality attributes, including CAR-T cell viability, T-cell differentiation state, exhaustion markers, and functional potency were evaluated in a good manufacturing practice (GMP)-compliant stability study up to 72 h. **Results:** For Jurkat cells, HPFs supplemented with SUL-138 and a combination of glucose, glutamine, and succinate demonstrated the greatest stability improvement at 2–8 °C, improving cell viability from ~1% to >85% after 72 h. For CAR-T cells, supplementation of HPFs with SUL-138 alone demonstrated the greatest improvement, resulting in a CAR-T cell viability from ~40% to >85% after 72 h of storage at 2–8 °C, while no additional benefits from mitochondrial substrates were observed. The novel HPF did not significantly impact CAR-T cell potency test results, T cell subset distribution, or exhaustion markers compared to control. **Conclusions:** A novel clinical-grade HPF that significantly improved fresh CAR-T cell stability during hypothermic storage was developed. This novel HPF can aid in the establishment of GMP-compliant and PoC CAR-T cell manufacturing platforms.

## 1. Introduction

CD19-targeted chimeric antigen receptor (CAR)-T cell therapy has yielded remarkable clinical responses in patients with relapsed or refractory B-cell malignancies, resulting in five Food and Drug Administration (FDA)-approved CAR-T cell drug products (DPs) for second- and third-line treatment [1,2,3,4,5,6,7,8,9,10,11,12,13,14,15,16,17]. Currently, all CAR-T cell DPs with a marketing authorization are manufactured at central manufacturing facilities, yielding a process that requires 3–6 weeks from starting material collection (apheresis material) to CAR-T cell administration to the patient (i.e., vein-to-vein time) [18,19,20,21]. Such prolonged turnaround times pose significant constraints for the timely and effective treatment of patients eligible for CAR-T therapy, particularly those with rapidly progressive disease. Furthermore, both the starting material and the final CAR-T cell DP are cryopreserved with dimethyl sulfoxide (DMSO). This requires shipment at ultra-low temperatures and exposing cells to two freeze–thaw cycles that negatively impact cell viability, and possibly CAR-T cell quality [22].

As an alternative, decentralized or Point-of-Care (PoC) manufacturing enables CAR-T cell production and treatment directly at clinical sites, thereby significantly reducing turnaround time and eliminating the need for cryopreservation. These fresh CAR-T cell DPs are typically stored in either simple aqueous solutions or complex hypothermic preservation formulations (HPFs) at 2–8 °C, while good manufacturing practice (GMP) procedures are completed for batch release. However, these procedures, including extensive quality control testing, must be completed in a timely manner since cells stored under hypothermic conditions exhibit a rapid decline in viability beyond 24 h [23]. Furthermore, patients may not be ready for infusion within this narrow 24 h window, e.g., due to transient fever after lymphodepletion that requires a delay in CAR-T cell infusion. Additionally, hypothermic stress may negatively impact critical quality attributes (CQAs) beyond viability, including potency and phenotype. These constraints highlight the need for a novel and optimized CAR-T cell HPF to improve stability and maintain product quality beyond 24 h. This would provide a broader window for completing GMP procedures as well as extend the shelf life of the CAR-T cell DP, enabling local/regional transportation and more flexible scheduling for administration to the patients.

A number of HPFs, such as the University of Wisconsin (UW) solution, Celsior, and histidine–tryptophan–ketoglutarate (HTK), have been developed for short-term hypothermic storage of cells. However, none of these HPFs sufficiently preserves cell viability beyond 48 h [24]. Although the HTS-FRS formulation has emerged as a modified medium capable of stabilizing cells for up to four days, its applicability has not yet been demonstrated for clinical-grade CAR-T cells manufactured under GMP conditions [25].

These commercially available HPFs are suboptimal in preventing hypothermic and rewarming-induced injury of the cells as a result of hypothermic storage at 2–8 °C, which is primarily caused by bioenergetic imbalance involving reactive oxygen species (ROS) accumulation and adenosine triphosphate (ATP) depletion [26,27,28,29]. Although hypothermia slows overall metabolic activity, cells still expend a basal level of energy for essential functions. This unmet energy demand causes ATP depletion, reduces enzymatic activity, triggers ion imbalance, and mitochondrial dysfunction [30,31,32,33,34,35]. Collectively, these disruptions lead to membrane depolarization, ROS accumulation, and ultimately apoptosis or necrosis, particularly upon rewarming [36,37,38]. Notably, this cascade of events forms a self-amplifying cycle, with impaired mitochondria producing excessive ROS that the weakened antioxidant systems cannot counteract, leading to further mitochondrial and cellular damage and eventual cell death. To mitigate these detrimental effects associated with hypothermic storage and rewarming, optimizing HPFs through the modulation of mitochondrial function is an attractive and feasible strategy [39].

Modulation of mitochondrial function can be achieved using so-called SUL-compounds that counteract hypothermia-induced cellular injury [40]. Their efficacy derives primarily from their capacity to stabilize mitochondrial membrane potential by activating mitochondrial membrane complexes I and IV, thereby maintaining ATP production and inhibiting ROS formation [41,42]. SUL-138, a compound in this class, has demonstrated protective effects across diverse disease models, including Alzheimer’s disease, acute kidney injury, and sepsis [41,42,43,44]. These beneficial effects are attributed to improving mitochondrial function, reducing inflammation, and preserving cellular integrity during hypothermic storage and subsequent rewarming.

In addition to SUL-138, metabolic substrates may support the cellular and mitochondrial function of cells during hypothermic storage. For example, glucose is typically present in HPFs to sustain cellular energy metabolism during hypothermic storage and subsequent rewarming [23]. In addition, glutamine supplementation has also been proposed, as it fuels biosynthesis and mitochondrial respiration via glutaminolysis, thereby producing tricarboxylic acid (TCA) cycle intermediates such as α-ketoglutarate that generate nicotinamide adenine dinucleotide (NADH) and flavin adenine dinucleotide (FADH_2_) [45,46]. Other substrates, such as succinate, can enhance oxidative phosphorylation by aiding in electron transport [47], whereas pyruvate and uridine improve mitochondrial respiration and redox balance. The latter has been demonstrated in zebrafish models of mitochondrial dysfunction and cardiac hypertrophy. Here, pyruvate enhanced respiratory function, and uridine reduced ROS [48]. Mitochondrial substrates intended for cell culture media are commercially available in pharmaceutical grade and are considered safe since these are endogenous metabolic substrates [49]. Therefore, these mitochondrial substrates are suitable as potential supplements for clinical-grade HPFs.

This study aimed to develop and optimize a novel HPF to improve the stability of fresh clinical-grade CAR-T cell DPs and extend their shelf life for at least 72 h. First, we screened a panel of novel HPFs supplemented with SUL-138 and/or mitochondrial substrates using the Jurkat cell line as model cells. From these experiments, HPFs were selected for further assessment using clinical-grade CD19 CAR-T cells. To assess the impact of potential temperature excursions during storage, we investigated two different storage conditions, namely 2–8 °C and 15–25 °C. CQA were assessed during a GMP-compliant stability study, such as viability, potency, exhaustion markers, and CAR-T cell phenotype, to ensure product quality and safety, as well as to obtain supporting data for the clinical applicability of the novel HPF.

## 2. Materials and Methods

### 2.1. Study Design

Jurkat cells and CAR-T cell DPs were evaluated under clinically relevant hypothermic conditions. Cells were formulated in in-house prepared CliniMACS Formulation Solution (CFS) [50] with or without 10 µM SUL-138 (±mitochondrial substrates: ~4.5 g/L D(+)-Glucose monohydrate (Merck, Darmstadt, Germany), ~584 mg/L L-glutamine (Thermo Fisher Scientific, Waltham, MA, USA), ~110 mg/L pyruvate (Sigma-Aldrich, St. Louis, MO, USA), ~1.2 g/L sodium succinate dibasic hexahydrate (Sigma-Aldrich, St. Louis, MO, USA), and ~50 mg/L uridine (Sigma-Aldrich, St. Louis, MO, USA). CFS alone refers to the in-house prepared base clinical formulation solution (control) without SUL-138 or mitochondrial substrate supplementation. Jurkat cells were formulated at a fixed density of 0.5 × 10^6^ cells/mL, whereas CAR-T cell DPs were formulated at either 2 × 10^6^ cells/mL or 0.5 × 10^5^ cells/mL and stored for up to 72 h at 2–8 °C or room temperature (RT;15–25 °C). After storage, cell viability, potency (defined by interferon gamma (IFN-γ) secretion), and phenotype (CD4/CD8 subset distribution, differentiation state, and exhaustion markers) were assessed according to a stability study plan and acceptance criteria set *a priori* to reflect the clinical applicability of the cell product after hypothermic storage (see Table S1). CAR-T cell DPs conditions were evaluated for three independent donors.

### 2.2. Cell Line and Culture

Jurkat clone E6-1 was obtained from the American Type Culture Collection (ATCC, Manassas, VA, USA) and cultured in RPMI Medium 1640 (Thermo Fisher Scientific, Waltham, MA, USA) supplemented with 10% fetal bovine serum (Sigma-Aldrich, St. Louis, MO, USA) and 1% penicillin-streptomycin (Thermo Fisher Scientific, Waltham, MA, USA) at 37 °C in a humidified atmosphere of 5% CO_2_ incubator.

### 2.3. CD19 CAR-T DP Production

CD19 CAR-T cell DPs were produced from apheresis material using the Clinimacs Prodigy (Miltenyi Biotec, Leiden, The Netherlands). Apheresis material was added to the CliniMACS Prodigy^®^ TS 520 tubing set (Miltenyi Biotec, Leiden, The Netherlands) and subsequently cells were selected for CD4 and CD8 using beads (CliniMACS^®^ CD4 Reagent CR/GMP and CliniMACS^®^ CD8 Reagent CR/GMP, Miltenyi Biotec, Leiden, The Netherlands) and CliniMACS PBS/EDTA buffer (Miltenyi Biotec, Leiden, The Netherlands) containing 0.5% human serum albumin (HSA) (Prothya Biosolutions Netherlands B.V., Amsterdam, The Netherlands). A total of 200 × 10^6^ CD3+ cells were seeded in the culture chamber and activated using MACS^®^ GMP T Cell TransAct™ Large Scale CR/GMP (Miltenyi Biotec, Leiden, The Netherlands) on the day of seeding. TexMACS™ GMP Medium (Miltenyi Biotec, Leiden, The Netherlands) containing 12.5 ng/mL MACS^®^ GMP Recombinant Human IL-7 (Miltenyi Biotec, Leiden, The Netherlands) and 12.5 ng/mL MACS^®^ GMP Recombinant Human IL-15 (Miltenyi Biotec, Leiden, The Netherlands) was used as the culture medium in the Clinimacs Prodigy. Viral transduction with CD19 CAR lentivirus was performed 16 h after activation. Culture wash and media exchange steps were performed starting 48 h after transduction. 7 days after seeding, cells were harvested, formulated, and used for experiments.

### 2.4. Preparation of Hypothermic Storage Solutions

CFS (Miltenyi Biotec, Bergisch Gladbach, Germany), an iso-osmotic salt solution supplemented with 2.5% HSA, was used as a formulation buffer. Components (Table S2) were dissolved in ultrapure water and mixed with HSA. pH was corrected to 6.9–7.0 with 1 M hydrochloric acid, and the solution was sterilized by filtration through a 0.2 µm diameter Whatman filter (Sigma-Aldrich, St. Louis, MO, USA).

SUL-138 (ROKEPIE, gifted by Sulfateq B.V., Groningen, The Netherlands) was used at 10 µM in all relevant conditions, based on a prior dose finding study (Figure S1). Mitochondrial substrates were added at concentrations corresponding to high-glucose basal cell culture media (~4.5 g/L glucose, ~584 mg/L glutamine) or based on data that support an increased stability of cells during hypothermic conditions (~1.2 g/L succinate, ~110 mg/L pyruvate, ~50 mg/L uridine) [51,52,53].

### 2.5. Short-Term Hypothermic Storage

Jurkat cells were seeded in 96-well plates (Corning Costar, Corning, NY, USA) at 1.0 × 10^6^ cells/mL in CFS or CFS supplemented with 10 µM of SUL-138 and combinations of glucose, glutamine, pyruvate, succinate, and uridine. Similarly, CD19 CAR-T cells were seeded in 96-well plates at densities of 2 × 10^6^ and 0.5 × 10^6^ cells/mL in identical formulations except pyruvate and uridine. The plates were stored at either 2–8 °C or RT for 72 h. All experiments were repeated at least three times.

### 2.6. Cell Viability Assay and Immunophenotyping

Viability of Jurkat cells was assessed using a FITC Annexin V Apoptosis Detection Kit with propidium iodide (PI) (BioLegend, San Diego, CA, USA). A staining solution was freshly prepared for each assessment by mixing FITC Annexin V, PI, Annexin V Binding Buffer, and H_2_O in a ratio of 1:1:32:46, respectively. Cells were incubated in the dark at room temperature for 15 min and measured on a NovoCyte Quanteon flow cytometer (Agilent, Santa Clara, CA, USA) within one hour of staining.

Following storage of CAR-T cells, samples were stained with FITC anti-CD3 (Miltenyi Biotec, Bergisch Gladbach, Germany) and 7AAD staining solution (Miltenyi Biotec, Bergisch Gladbach, Germany) to assess cell viability. Cells were incubated at 2–8 °C in the dark for 20 min. Samples were acquired in a MACSQuant Analyzer 10 (Miltenyi Biotec, Bergisch Gladbach, Germany) and data were analyzed using FlowJo software 10.0 (FlowJo LLC, Ashland, OR, USA).

The following antibodies were used for characterization of T cell subsets: Zombie Red (BioLegend, San Diego, CA, USA), Pacific Blue anti-CD3 (BD Biosciences, BD Biosciences, San Jose, CA, USA), Spark YG 581 anti-CD4 (BioLegend, San Diego, CA, USA), BUV805 anti-CD8 (BD Biosciences, San Jose, CA, USA), BV480 anti-CD45RO (BD Biosciences, BD Biosciences, San Jose, CA, USA), BUV615 anti-CD45RA (BD Biosciences, BD Biosciences, San Jose, CA, USA), APC anti-CD62L (BD Biosciences, San Jose, CA, USA), PE-Fire 810 anti-CCR7 (BioLegend, San Diego, CA, USA), BUV737 anti-CD95 (BD Biosciences, BD Biosciences, San Jose, CA, USA), StarBright UV445 anti-CD25 (Bio-Rad Laboratories, Kidlington, UK), Real Blue 744 anti-CD27 (BioLegend, San Diego, CA, USA), BV750 anti-CD69 (Bio-Rad Laboratories, Kidlington, UK), BV750 anti-CD279 (PD-1) (BD Biosciences BD Biosciences, San Jose, CA, USA), BV605 anti-CD366 (TIM-3) (BD Biosciences, BD Biosciences, San Jose, CA, USA), APC-Cy7 anti-CD223 (LAG3) (BioLegend, San Diego, CA, USA), PE-Fire 810 anti-CD39 (BioLegend, San Diego, CA, USA) and Brilliant Blue 700 anti-CD152 (CTLA-4) (BioLegend, San Diego, CA, USA). Cells (1.0 × 10^5^) were washed and re-suspended in 100 µL of BD Horizon Brilliant Stain Buffer (BD Biosciences, San Jose, CA, USA) containing the antibodies mentioned above, incubated for 30 min at 2–8 °C, washed three times, and measured with FACSymphony A5 (BD Biosciences, San Jose, CA, USA). Samples were analyzed by NovoExpress software 1.6.1 (Agilent, Santa Clara, CA, USA).

### 2.7. Secretion of IFN-γ (Potency Assay)

To assess the potency of the CAR-T cells, a fully GMP-validated potency assay was used. IFN-γ secretion upon antigen-specific activation of CD19 CAR-T cells stored under hypothermic conditions for 72 h was measured after a 24 h incubation period of CD19 CAR-T cells cultured with Dynabeads M-280 Streptavidin (Thermo Fisher Scientific, Waltham, MA, USA) loaded with recombinant human CD19 antigen, i.e., CD19 CAR detection reagent (Miltenyi Biotec, Leiden, The Netherlands). Culture of CAR-T cells and Dynabeads was performed with a cell-to-bead ratio of 1:10 (5 × 10^4^:5 × 10^5^ cells) in RPMI 1640 medium (Gibco, Thermo Fisher Scientific, Waltham, MA, USA) supplemented with 10% FBS (Sigma-Aldrich, St. Louis, MO, USA) in a 37 °C, 5% CO_2_ humidified incubator. Dynabeads not loaded with CD19 CAR detection reagent but incubated in PBS (Gibco, Thermo Fisher Scientific, Waltham, MA, USA) only served as the negative control. Culture supernatants were collected directly after the 24 h incubation period, and sample preparation was conducted according to the protocol for the Simple Plex Human IFN-gamma (3rd Gen) Cartridge (Bio-Techne, Minneapolis, MN, USA). The potency result was determined by subtracting the non-loaded bead potency test value (negative control) from the CD19-loaded bead potency test value obtained after 24 h of co-culture with CAR-T cells.

### 2.8. Statistical Analysis

All averaged values represent mean ± SD from at least 3 independent experiments. Differences between groups were assessed using an unpaired two-tailed Student’s *t*-test, a Mann–Whitney U-test, or one-way ANOVA followed by Tukey’s multiple-comparison test, where appropriate. Statistical analyses were performed using GraphPad Prism (version 9.1.0 or 10.2.3, La Jolla, CA, USA). More details on statistical analysis are outlined in the Supplementary Materials (Table S3).

## 3. Results

### 3.1. HPF Supplemented with SUL-138 Improves Viability of Jurkat Cells Stored Under Hypothermic Conditions

**Figure 1:**
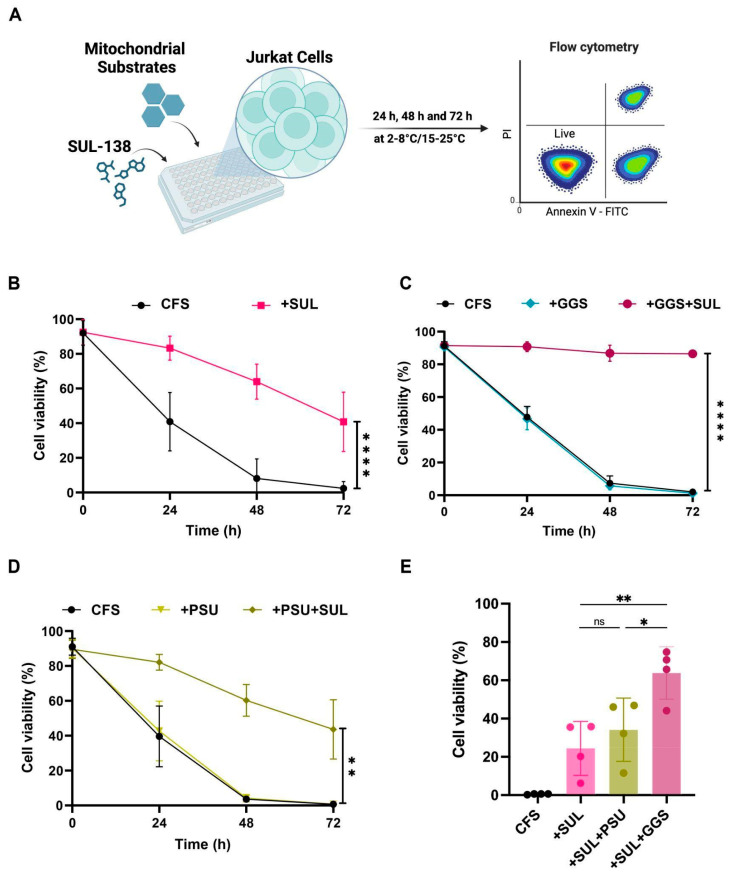
SUL–138 improves the viability of Jurkat cells stored in standard CliniMACS Formulation Solution (CFS) or CFS supplemented with mitochondrial substrates at 2–8 °C. (**A**) Schematic illustration of experimental scheme of hypothermic preservation formulation (HPF) screening for improving cell viability during hypothermic storage based on the proportion of AnnexinV-PI- subset in Jurkat cells stored at 2–8 °C for 72 h (figure created using Biorender (https://biorender.com/; accessed on 2 February 2026). (**B**–**D**) Viability of Jurkat cells stored in SUL–138-supplemented CFS with the addition of mitochondrial substrates. (**E**) Bar graph of Jurkat viability in HPF formulations after 72 h incubation. * *p* < 0.0332, ** *p* < 0.0021, *** *p* < 0.0002, and **** *p* < 0.0001, ns, not significant; by Mann–Whitney U-Test (**B**) or unpaired two-tailed Student’s *t*-test (**C**–**E**). GGS—a combination of glucose, glutamine, and succinate. PSU, a combination of pyruvate, succinate, and uridine.

In an initial screen of HPFs to assess the stability of Jurkat cells (Figure 1A), hypothermic storage at 2–8 °C in standard CFS was found to rapidly decrease Jurkat viability to 41% at 24 h, 8% at 48 h, and 2% at 72 h (Figure 1B). Notably, the addition of SUL alone to CFS already improved Jurkat viability to 83% at 24 h, 64% at 48 h, and 41% at 72 h. In contrast, supplementing CFS with only mitochondrial substrates did not significantly improve the viability of Jurkat cells (Figure 1C,D). However, adding SUL-138 to CFS with glucose, glutamine, and succinate (GGS) significantly improved viability from 47% to 91% at 24 h, from 6% to 87% at 48 h, and from 1% to 86% at 72 h. When SUL-138 was added to CFS supplemented with pyruvate, succinate, and uridine (PSU), viability improved from 43% to 82% after 24 h, from 4% to 60% after 48 h, and from 1% to 44% after 72 h. Notably, the combination of SUL and GGS was superior to only SUL-138 or a combination of SUL-138 and PSU, with a viability at 72 h of 86% vs. 41% and 44%, respectively (Figure 1E). Of note, this stability-improving effect was smaller when Jurkat cells were stored at RT, where SUL and GGS only increased viability to 62% at 72 h (Figure S2).

Taken together, SUL-138 supplementation significantly improved the viability of Jurkat cells stored at 2–8 °C for up to 72 h, with the greatest effect observed when combined with GGS.

### 3.2. SUL-138 Improves the Stability of Fresh CAR-T Cell DPs Stored Under Hypothermic Conditions

**Figure 2:**
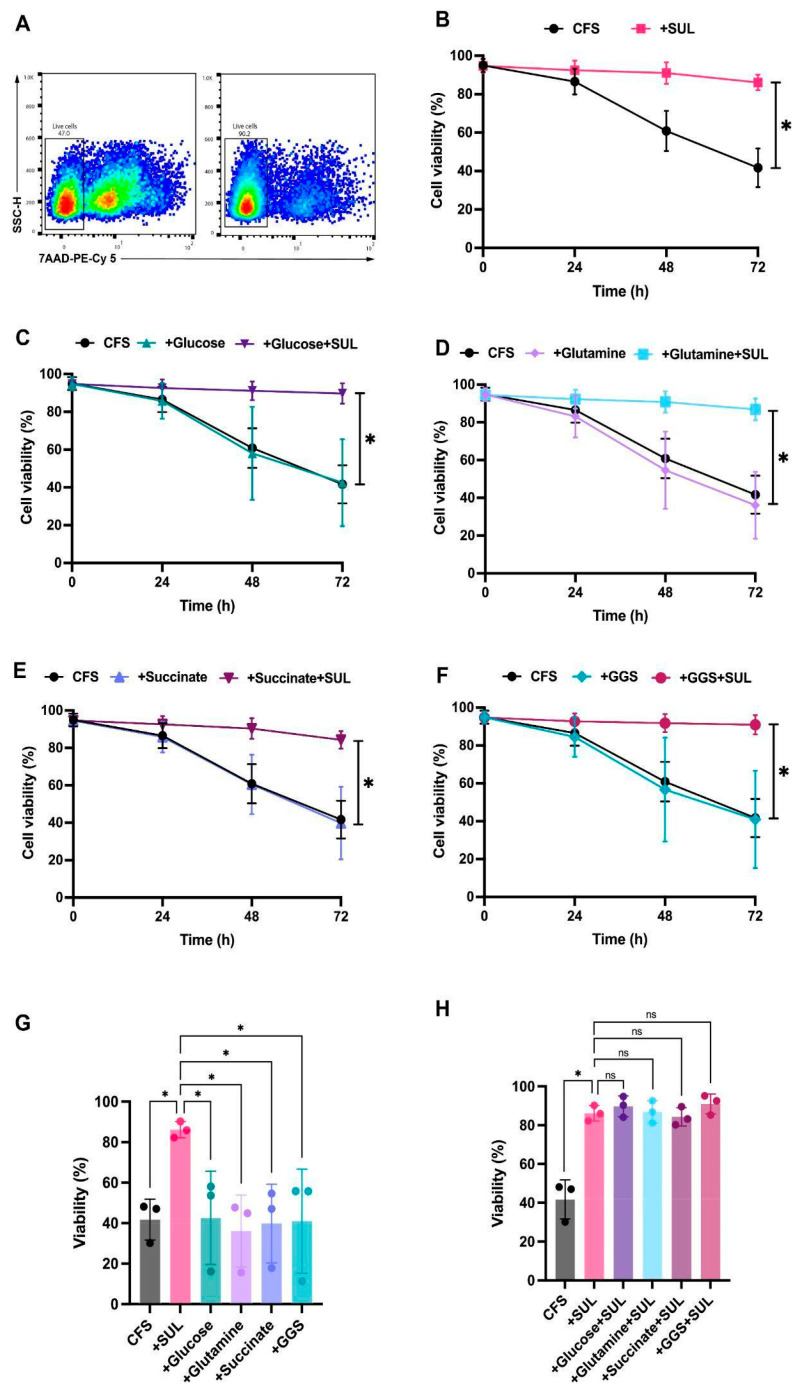
CFS supplemented with SUL-138 improves the stability of clinical-grade, fresh CD19 chimeric antigen receptor (CAR)-T cell drug products (DPs) stored at 2–8 °C. Illustrative flow cytometry plots of CAR-T DPs after 72 h storage in CFS (left) or CFS supplemented with SUL-138 and GGS (right). (**A**) Percentage of viability (7AAD-) of CD19 CAR-T DPs stored with CFS and CFS + SUL (**B**), CFS, Glucose and Glucose + SUL (**C**), CFS, Glutamine and Glutamine + SUL (**D**), CFS, Succinate and Succinate + SUL (**E**), and CFS, GGS and GGS + SUL (**F**) for 72 h. Comparison of cell viability (%) after storage in CFS supplemented with SUL and CFS supplemented with mitochondrial substrates alone. (**G**) and mitochondrial substrates combination with SUL (**H**) at 72 h. * *p* < 0.0332, ** *p* < 0.0021, *** *p* < 0.0002, and **** *p* < 0.0001; ns, not significant by a one-way analysis of variance (ANOVA) with Tukey’s multiple-comparison test. GGS—a combination of glucose, glutamine, and succinate.

At 2–8 °C, the HPFs supplemented with either SUL-138 alone or in combination with GGS were the only formulations that significantly improved the stability of CD19 CAR-T cells after 72 h of storage compared to CFS (Figure 2A). Specifically, viability reached 86% with SUL-138 alone, which was not significantly improved by supplementation with either glucose (90%), glutamine (87%), or succinate (84%) (Figure 2B–F) or a combination of mitochondrial substrates (Figure 2H). Similarly, stability was not improved by adding mitochondrial substrates either individually or in combination with the SUL-138 (Figure 2G–H).

This stability-improving effect was not observed when cells were stored at RT for 72 h (Figure S3). Under these conditions, SUL-138 alone did not improve viability, but viability was significantly improved when combined with the three mitochondrial substrates GGS (76% vs. 42%; Figure S3I,J). Additionally, viability reached 70% with glucose supplementation alone at RT, but neither glutamine nor succinate significantly improved viability (Figure S3). Taken together, SUL-138 significantly improved the viability of CAR-T cells stored at 2–8 °C, but not stored at RT. Furthermore, supplementation of mitochondrial substrates improved the viability of CAR-T cells stored at RT.

Beyond the effects of the constituents of the HPFs, we also evaluated the impact of cell density on CAR-T cell viability by testing two CAR-T cell concentrations (2 × 10^6^ and 0.5 × 10^6^ cells/mL) during the 72 h storage period at 2–8 °C. CAR-T cell viability did not differ between the two tested concentrations during the 72 h storage period at 2–8 °C. (Figure S4). During storage at RT, however, viability was consistently higher at 2 × 10^6^ cells/mL than at 0.5 × 10^6^ cells/mL, with a difference of 15% across all tested formulations (Figure S3).

### 3.3. HPFs Preserve CAR-T Cell Phenotype and Functional Potency During Hypothermic Storage

**Figure 3:**
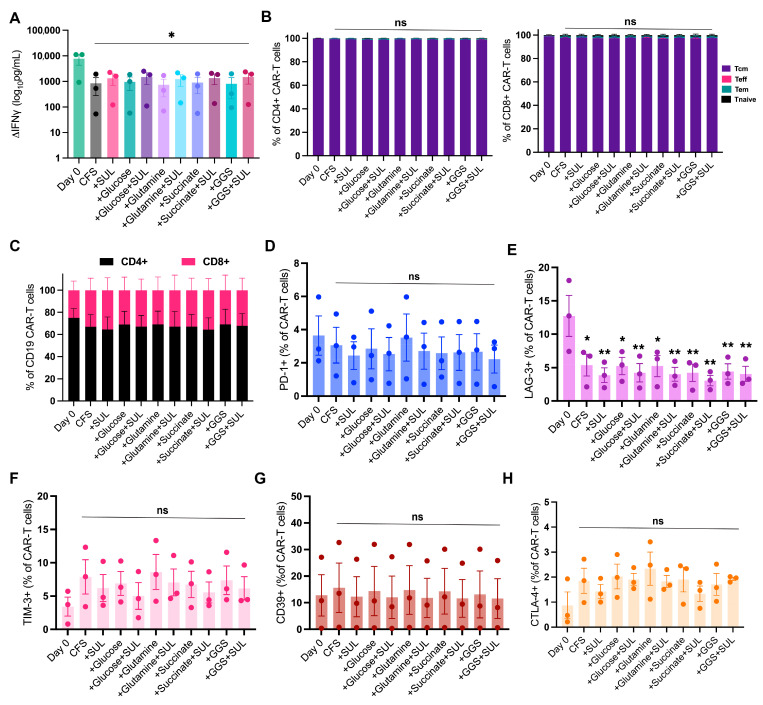
CAR-T DPs stored in CFS supplemented with SUL-138 and mitochondrial substrates remain potent with no significant shift in phenotypical subsets during hypothermic storage for 72 h. Interferon gamma (IFN-γ) secretion of CAR-T cell DPs following 72 h of hypothermic storage relative to the freshly harvested DPs when co-cultured with CD19-loaded beads for 24 h. ΔIFN-γ difference relative to non-loaded beads (negative control). (**A**) Proportion of central memory (CCR7 ^+^, CD45RO^+^), effector (CCR7^−^, CD45RO^−^), effector memory (CCR7^−^, CD45RO^+^), and naive (CCR7+CD45RO-) subsets. (**B**) Flow cytometric analysis of the CD4^+^/CD8^+^ CAR-T DPs ratio. (**C**) Percentage of CAR-T cell DPs expressing the inhibitory receptors PD-1 (**D**), LAG-3 (**E**), TIM-3 (**F**), CD39 (**G**), and CTLA-4 (**H**). * *p* < 0.0332, ** *p* < 0.0021, *** *p* < 0.0002, and **** *p* < 0.0001; ns, not significant by a one-way analysis of variance (ANOVA) with Tukey’s multiple-comparison test.

In addition to viability, CAR-T cell potency and phenotype are CQA of the DP. Therefore, these CQAs were assessed during the stability study. For CAR-T cell potency assessment, IFN-γ secretion upon antigen-specific (CD19) activation of the CAR-T cells was tested as a surrogate potency marker. IFN-γ secretion upon antigen-specific CAR-T cell activation significantly decreased after 72 h of storage in all tested HPFs compared to the freshly harvested CAR-T cells. When each donor was checked separately, two of the three donors exceeded the initial batch release acceptance criterion of ≥300 pg/mL IFN-γ, whereas one donor remained below this threshold (97 +/− 35 pg/mL; Figure 3A). Notably, this specific donor exhibited lower cytokine secretion than the other two donors and expressed higher levels of exhaustion markers (Figure 3D–H), suggesting that the low secretion was donor-specific. Despite donor variation, all three CAR-T cell DPs tested were fully functional and potent, demonstrating a significant IFN-γ secretion upon CD19 antigen-specific stimulation that did not differ from the control group, and thereby, complying with the end-of-shelf-life specification (Table S1). Across all formulations, supplementation of standard CSF with either SUL-138 or mitochondrial substrates did not significantly impact the potency test results of the CAR-T cell DPs.

Clinical outcomes are associated with the functionality and the phenotype of CAR-T cell DPs and are therefore critical to evaluate [54,55,56,57,58]. Hypothermic storage did not significantly alter CD4/CD8 frequencies or the distribution of memory and effector subsets in any tested HPF (Figure 3C). Specifically, the proportions of central memory (Tcm), effector (Teff), effector memory (Tem), and naïve (Tn) T cells did not alter within either CD4^+^ or CD8^+^ populations across all tested HPFs (Figure 3B,C). Furthermore, the expression of exhaustion markers, including PD-1, TIM-3, CD39, and CTLA-4, was not significantly impacted by hypothermic storage compared to freshly harvested CAR-T cells (Figure 3E–H). However, LAG-3 expression declined significantly from 13% to 4% (Figure 3D), but this was considered acceptable (Table S1).

In conclusion, the phenotypic subsets and exhaustion marker expression of the CAR-T cell DPs were not significantly impacted compared to freshly formulated CAR-T cells after 72 h of hypothermic storage. While donor-to-donor variation was evident in the potency assay, all CAR-T cell DPs remained potent and functional, and the potency test results were not significantly impacted compared to the standard CFS control group (Table S1). Since SUL-138 supplementation improved CAR-T cell DP stability (viability >80%) and preserved CQAs, and no additional benefits were observed with mitochondrial substrate supplementation, the HPF supplemented with SUL-138 alone was selected as the novel and optimal HPF for fresh CAR-T cells DPs.

## 4. Discussion

This study presents a novel HPF containing SUL-138 that significantly improves the stability of clinical-grade CD19 CAR-T cell DPs stored under hypothermic conditions (2–8 °C) without impacting CAR-T cell potency test results or phenotype. Since the safety of SUL-138 has been shown in preclinical studies and a phase I clinical trial [43,59,60,61], the safety of the novel HPF is substantiated and the present study provides supporting data of its use in clinical practice. The ability to extend the shelf life of CAR-T cell DPs enables greater flexibility for quality control testing, batch release procedures, patient planning, and transport, thereby aiding in the establishment of GMP-compliant PoC manufacturing platforms. To our knowledge, this is the first report presenting the development of a clinical-grade HPF that significantly improves the stability of fresh CAR-T cell DPs.

CAR-T cell viability after 72 h of storage at 2–8 °C with SUL-138 was consistently high (86%), exceeding the ≥70% viability acceptance criterion required for clinical release of CAR-T cell DPs. By contrast, mitochondrial substrates, either alone or in combination, did not improve the viability during hypothermic storage, but rather improved the viability during storage at RT. This difference likely results from the suppression of basal metabolic rates at low temperatures (2–8 °C), which limits the cellular demand for and utilization of these supplemented substrates. In addition, the stability-improving effect of SUL-138 was more pronounced at a lower temperature (2–8 °C) than at RT, which is consistent with the previous reports of SUL derivatives that support mitochondrial activity and maintain ATP production under hypothermic conditions [40,41,42,62,63]. Preservation of viability by SUL-138 was consistent across both cell concentrations tested (2 × 10^6^ and 5 × 10^5^ cells/mL), which were the cell concentration ranges in our clinical-grade CAR-T cell DPs, supporting the clinical applicability of the developed HPF.

While SUL-138 supplementation ensures stability and high CAR-T cell viability, preserving CAR-T cell functionality is equally critical to meet acceptance criteria for a stable and clinical-grade DP. IFN-γ secretion upon antigen-specific CAR-T cell activation was significantly lower in hypothermically stored CAR-T cell DPs compared to freshly harvested CAR-T DPs, suggesting that the decrease across all formulations might be a cold-induced effect. Low temperatures slow energy-dependent processes such as protein synthesis, which account for the reduced cytokine production following hypothermic storage. Consequently, it may be necessary to rewarm and transiently rest [64] hypothermically stored CAR-T cells prior to potency testing to allow cells for metabolic recovery and ensure a more accurate comparison with freshly harvested CAR-T cells.

Since CAR-T cell phenotypical subsets (e.g., Tn, Tcm) and exhaustion markers correlate with clinical outcomes [54,55,56,57,58], these were analyzed during the stability study to ensure no significant undesired changes occurred. The CD4/CD8 frequency and phenotypical subsets of the CAR-T cell DPs were not impacted during storage in all tested HPFs. Despite marked differences in viability depending on the formulation, the relative composition of these populations was not impacted by hypothermic storage for 72 h. Furthermore, although the expression of exhaustion markers varied widely across donors, no significant undesired increase in exhaustion markers was observed during the entire hypothermic storage period of all tested HPFs, which demonstrated no undesired shifts during the hypothermic storage period.

While cell lines are advantageous for initial buffer development, a clear difference in response to hypothermic storage was observed here between Jurkat and primary CD19 CAR-T cells. Jurkat cells exhibited greater sensitivity to hypothermic stress than primary CAR-T cells, likely because hypothermia affects cell types based on their specific metabolic demands. Jurkat cell line—derived from human acute T cell leukemia—consists of rapidly proliferating cells that have a high metabolic demand and rely heavily on mitochondria-independent glycolysis, making them more prone to rapid ATP depletion and lactate-induced acidosis during hypothermic storage [65,66,67]. By contrast, CAR-T cells are derived from primary cells and do not proliferate readily without activating stimuli. In addition, CAR-T cells in this study consisted primarily of central memory T cells, which preferentially rely on mitochondria-dependent oxidative phosphorylation. This reliance on oxidative metabolism may preserve mitochondrial function and make CAR-T cells less susceptible to hypothermia-induced mitochondrial damage during hypothermic storage [66,68,69,70].

The safety and toxicity of novel excipients like SUL-138 are pivotal for clinical application in HPFs, such as HPFs for clinical-grade CAR-T cells. In preclinical studies, SUL-138 demonstrated an absence of mutagenicity and genotoxicity [43], while 30-day oral repeated-dose toxicity studies in rats and minipigs showed no adverse effects on complete blood cell counts or hematopoietic and lymphoid organs [59]. No Observed Adverse Effect Levels (NOAELs) were established in these models (150 and 90 mg/kg/day, respectively), with corresponding maximum plasma concentration (C_max_) values exceeding 4.7 × 10^3^ µg/L in rats and 2.0 × 10^3^ µg/L in minipigs. The 10 µM concentration used in our HPF (equivalent to 3.6 × 10^3^ µg/L) falls within the same range of these preclinical exposure levels at NOAEL. Furthermore, since CAR-T cell formulations are typically administered in volumes of 30–100 mL and diluted by the blood volume (~5 L) of the patient upon slow infusion, this dosage provides a safety margin of at least 45-fold relative to nominal blood volumes [60]. Most importantly, a recent phase I study confirmed that SUL-138 was well tolerated after single oral doses up to 2000 mg and repeated doses of 2000 mg b.i.d. and 1500 mg t.i.d. for 14 days [61]. Consequently, a single infusion of SUL-138-supplemented CAR-T cell HPF is expected to be well tolerated in clinical practice, and the safety is substantiated by ample preclinical and clinical safety/toxicity data.

The literature regarding the storage of cellular products of human origin is limited, as most efforts have focused on the cryopreservation of cellular drug products [22,71,72]. Although CAR-T cell stability in HPFs has not been described previously, conventional HPFs typically preserve viability for only 24 to 48 h. For instance, standard solutions like UW and Ringer’s often failed to maintain viability above 40% in various primary cell models after 48–72 h storage [36,73]. Even specialized formulations such as HypoThermosol FRS (HTS-FRS) show highly variable performance depending on the cell type; while it preserved MSC viability at ~80% viability after 4 days of storage [25,74], it yielded only 67% for human hepatocytes after 72 h of storage [75]. In our study, the viability of CAR-T cells was ~86% after 72 h when stored in SUL-138-supplemented HPF, outperforming conventional HPFs and demonstrating compliance with clinical-grade acceptance criteria for viability, potency, phenotypical subsets, and exhaustion markers.

Although CQAs of clinical-grade CAR-T cells were extensively assessed in a GMP-compliant stability study, several limitations of the present study remain. First, to assess the potency of the CAR-T cells, an IFN-γ release potency assay was used, which is a surrogate potency assay. The cytotoxic potency, repeated challenge, or in vivo efficacy of the CAR-T cells were not assessed. Furthermore, the metabolic characteristics as well as mitochondrial function of the CAR-T cells stored under hypothermic conditions in the novel HPF were not assessed. It is, therefore, currently unclear whether the cytotoxic potency, persistence, mitochondrial function, metabolic characteristics, and possibly in vivo efficacy of CAR-T cells stored under hypothermic conditions in the novel HPF are impacted. These analyses should be performed in future research.

In conclusion, CFS supplemented with SUL-138 was selected as the optimal and novel HPF for clinical translation that significantly improved CD19 CAR-T cell DP stability. Since mitochondrial substrates confer no additional benefit, excluding them simplifies the formulation, making it less laborious and cheaper to manufacture while reducing potential instability issues of the constituents. Since standard CFS is already of clinical-grade quality and the safety of SUL-138 has been demonstrated, the present study provides supporting data for the use of the novel HPF in clinical practice.

## Data Availability

Data can be made available upon request.

## Supplementary Materials

The following supporting information can be downloaded at: https://www.mdpi.com/article/10.3390/pharmaceutics18040414/s1, Table S1: Fresh CAR-T cell drug product stability study plan and acceptance criteria; Table S2: Constituents of in-house prepared CFS; Figure S1: SUL-238 maximally improves Jurkat cell viability at 10 µM, with no further improvement by increased doses; Table S3: Statistical analysis; Figure S2: SUL-238 improves cell viability of Jurkat cells stored in standard HPF or HPFs supplemented with mitochondrial substrates at RT; Figure S3: Viability of CAR-T drug products decreased over 24 h of storage at RT; this decline was partially mitigated by mitochondrial substrate supplementation; Figure S4: SUL-238 improves CAR-T cell viability during 72 h storage at 2–8 °C, regardless of mitochondrial substrate supplementation or cell concentration.

## Abbreviations

The following abbreviations are used in this manuscript:

CAR	Chimeric antigen receptor
FDA	Food and Drug Administration
HPF	Hypothermic preservation formulation
DP	Drug product
GMP	Good manufacturing practice
DMSO	Dimethyl sulfoxide
PoC	Point-of-care
CQA	Critical quality attribute
UW	University of Wisconsin
HTK	Histidine-tryptophan-ketoglutarate
HTS	HypoThermosol
ROS	Reactive oxygen species
ATP	Adenosine triphosphate
TCA	Tricarboxylic acid
NADH	Nicotinamide adenine dinucleotide
FADH_2_	Flavin adenine dinucleotide
RT	Room temperature
GGS	Glucose, glutamine, succinate
PSU	Pyruvate, uridine, succinate
CFS	CliniMACS Formulation Solution
TCA	Tricyclic acid
HSA	Human serum albumin
PI	Propidium iodide
ANOVA	Analysis of variance
Tcm	Central memory T cell
Teff	Effector T cell
Tem	Effector memory T cell
Tn	Naïve T cell
NOAEL	No observed adverse effect level
