# Supplementary material for: A Novel Hypothermic Preservation Formulation Containing SUL-138 Enables Long-Term Hypothermic Storage of Clinical-Grade CAR-T Cells
Source: Pharmaceutics. 2026 Mar 28;18(4):414. doi: 10.3390/pharmaceutics18040414 (PMC13118681; doi:10.3390/pharmaceutics18040414)
Supplement: Supplementary file 1 [file pharmaceutics-18-00414-s001.zip › pharmaceutics-4190735-supplementary.pdf]

Supplementary Materials

Table S1. Fresh CAR-T cell drug product stability study plan and acceptance criteria.

Specification	Method	T=0 (Day 0)	T=24 h	T=48 h	T=72 h
Viability	Flow cytometry	≥70%	≥70%	≥70%	≥70%
Potency	IFN-γ secretion (bead-based assay)	≥300 pg/ml	n.t.	n.t.	Significant increase in IFN-y secretion upon CD19 antigen-specific stimulation<br><br>AND<br><br>No significant difference in potency test results compared to CAR-T cells formulated in standard CFS (control group)
Exhaustion markers	Flow cytometry	For information	n.t.	n.t.	No significant increase in exhaustion marked
Phenotypical subsets	Flow cytometry	For information	n.t.	n.t.	No significant change vs. T = 0 h

Abbreviations: n.t., not tested; IFN-γ, interferon-gamma. "For information" indicates parameters recorded for characterization only and not applied as formal acceptance criteria.

Table S2. Constituents of in-house prepared CFS.

Constituent	Amount per 100 mL of CFS	Supplier (Location)
**Sodium chloride**	0.473 g	Merck (Darmstadt, Germany)
**Potassium chloride**	0.034 g	Merck (Darmstadt, Germany)
**Magnesium chloride hexahydrate**	0.028 g	Merck (Darmstadt, Germany)
**Sodium acetate trihydrate**	0.331 g	Sigma-Aldrich (St. Louis, MO, USA)
**Sodium gluconate**	0.452 g	Sigma-Aldrich (St. Louis, MO, USA)
**Tri sodium citrate dihydrate**	0.29 g	Sigma-Aldrich (St. Louis, MO, USA)
**Human serum albumin 20%**	12.5 mL	CSL Behring (King of Prussia, PA, USA)
**Ultrapure water**	87.5 mL	Merck Milli-Q (Darmstadt, Germany)
**Hydrochloric acid 1M**	q.s.	Merck (Darmstadt, Germany)

Abbreviations: q.s., quantum satis: added as needed to adjust pH to 6,9-7,0.

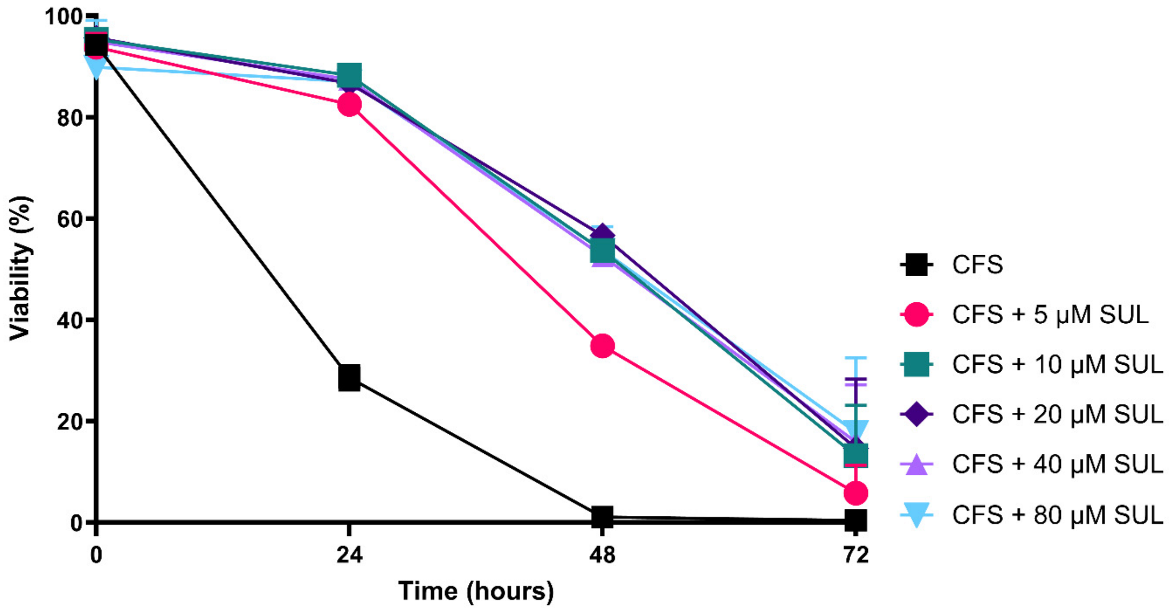

Figure S1. SUL-138 maximally improves Jurkat cell viability at 10 μM, with no further improvement by increased doses. Cells were stored in CFS supplemented with 5, 10, 20, 40 or 80 μM of SUL-138 at 2-8 °C for 72 hours. Data are represented as mean ± SD of n=2 experiments.

Table S3. Statistical analysis

Figure no.	Symbols	Statistical Test	Adjusted P value
1	B	Mann-Whitney Test	72h: CFS vs CFS+SUL p<0.0001
	C	Unpaired T-Test	72h: GGS vs. GGS+SUL p<0.0001
			72h: CFS vs GGS ns
	D	Unpaired T-Test	72h: +PSU vs. +SUL+PSU p = 0.0024
			72h: CFS vs. +PSU ns
	E	Unpaired T-Test	72h: +SUL vs. +SUL+GGS p=0.0071
			72h: +SUL vs. +SUL+PSU ns
			72h: +SUL+PSU vs. +SUL+GGS p=0.0326
2	B,C,D,E,F	One-way ANOVA	72h: CFS vs CFS p= 0.0332
		Tukey post hoc test	CFS+Glucose vs CFS+Glucose+SUL p= 0.0202
			CFS+Glutamine vs CFS+Glutamine+SUL p=0.0105
			CFS+Succinate vs CFS+Succinate+SUL p= 0.0324
			CFS+GGS vs CFS+GGS+SUL p=0.0120
3	A	One-way ANOVA	72h: All conditions ns
		Dunnett's multiple comparisons test	
	B(left)	One-way ANOVA	72h: All conditions ns
		Dunnett's multiple comparisons test	
	B(right)	One-way ANOVA	72h: All conditions ns
		Dunnett's multiple comparisons test	
	C	One-way ANOVA	72h: All conditions ns
		Dunnett's multiple comparisons test	
	D	One-way ANOVA	72h:
		Dunnett's multiple comparisons test	Day 0 vs. CFS p= 0,0178
			Day 0 vs. +SUL p=0.0036
			Day 0 vs. +Glucose p=0.0155
			Day 0 vs. +Glucose+SUL p=0.0053
			Day 0 vs. +Glutamine p=0.0156

		Day 0 vs. +Glutamine+SUL p=0.0041<br>Day 0 vs. +Succinate p=0.0053<br>Day 0 vs. +Succinate+SUL p=0.0015<br>Day 0 vs. +GGS p=0.0065<br>Day 0 vs. +GGS+SUL p=0.0044
E	One-way ANOVA<br>Dunnett's multiple comparisons test	72h: All conditions ns
F	One-way ANOVA<br>Dunnett's multiple comparisons test	72h: All conditions ns
G	One-way ANOVA<br>Dunnett's multiple comparisons test	72h: All conditions ns
H	One-way ANOVA<br>Tukey post hoc test	72h:<br>Day 0 vs. CFS p= 0.0127<br>Day 0 vs. +SUL p=0.0235<br>Day 0 vs. +Glucose p=0.0149<br>Day 0 vs. +Glucose+SUL p=0.0272<br>Day 0 vs. +Glutamine p=0.0108<br>Day 0 vs. +Glutamine+SUL p=0.0201<br>Day 0 vs. +Succinate p=0.0135<br>Day 0 vs. +Succinate+SUL p=0.0238<br>Day 0 vs. +GGS p=0.0120<br>Day 0 vs. +GGS+SUL p=0.0274<br>Other conditions: ns

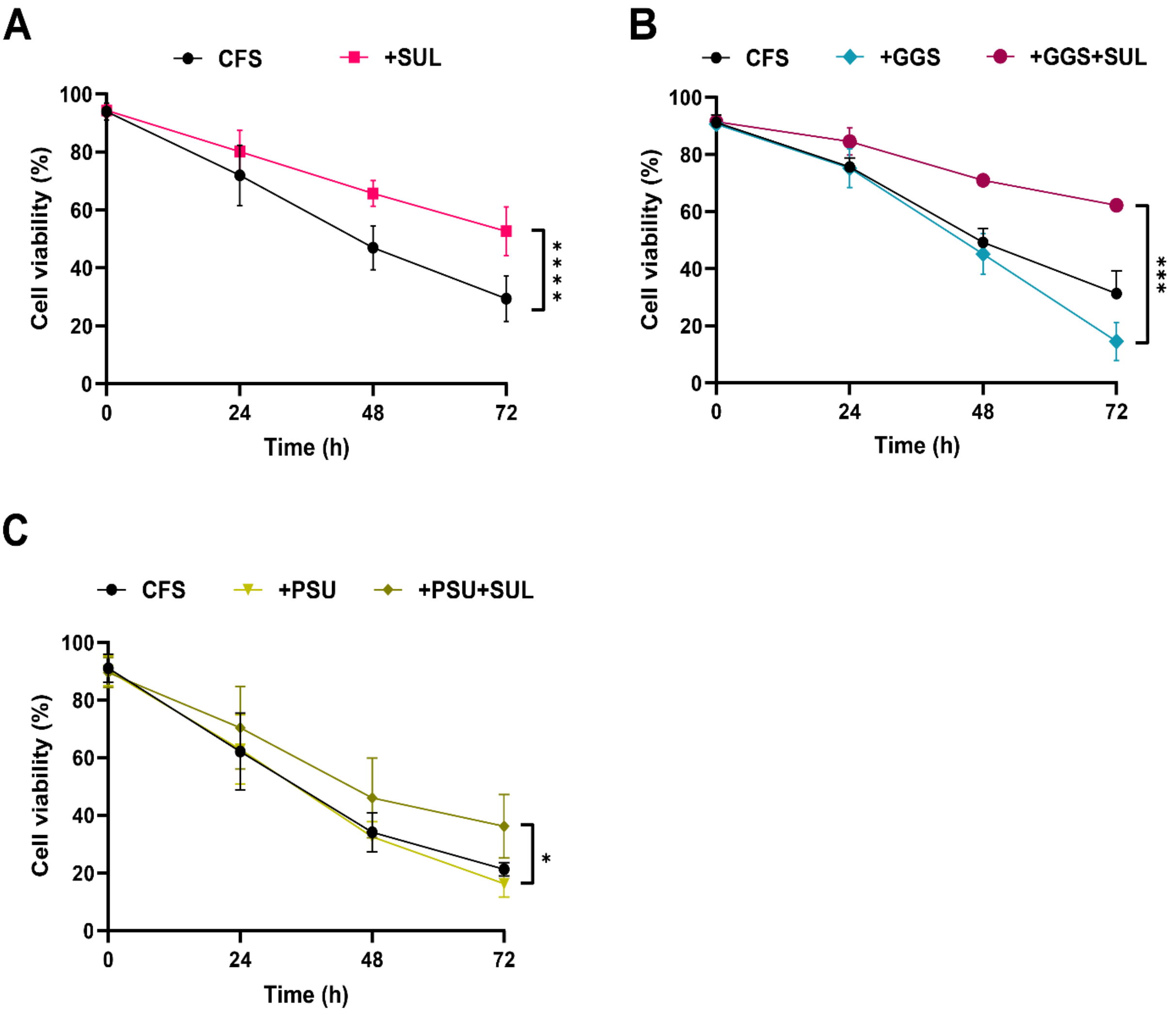

Figure S2. SUL-138 improves cell viability of Jurkat cells stored in standard HPF or HPFs supplemented with mitochondrial substrates at RT. Data are presented as the mean values ± SD of n= 3-11 independent experiments. *p < 0.0332, **p < 0.0021, ***p < 0.0002, and ****p < 0.0001, ns, not significant; by unpaired two-tailed Student's t-test (A, B) or Mann-Whitney Test (C). GGS, combination of glucose, glutamine, and succinate. PSU, combination of pyruvate, succinate and uridine.

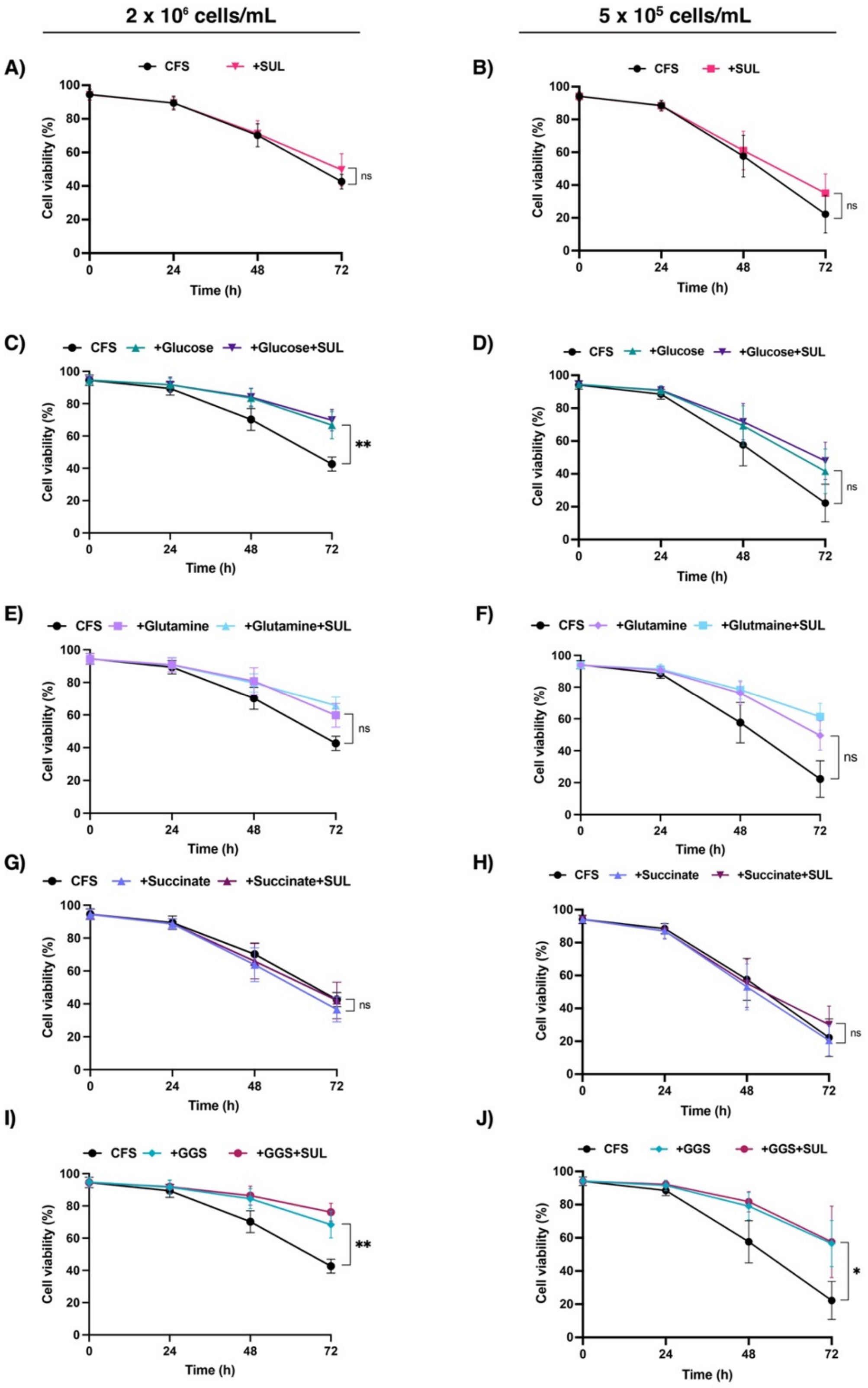

Figure S3. Viability of CAR-T drug products decreased over 24 h of storage at RT; this decline was partially mitigated by mitochondrial substrate supplementation. Slightly higher viability was observed at the higher cell concentration (2 × 10^6 cells/mL) compared with the lower concentration (5 × 10^5 cells/mL). (A, C, E, G, I) Viability at 2 × 10^6 cells/mL. (B, D, F, H, J) Viability at 5 × 10^5 cells/mL. Data are mean ± SD of n = 4 biologically independent samples. ns, not significant by one-way ANOVA with Tukey's multiple-comparison test. GGS: combination of glucose, glutamine, and succinate.

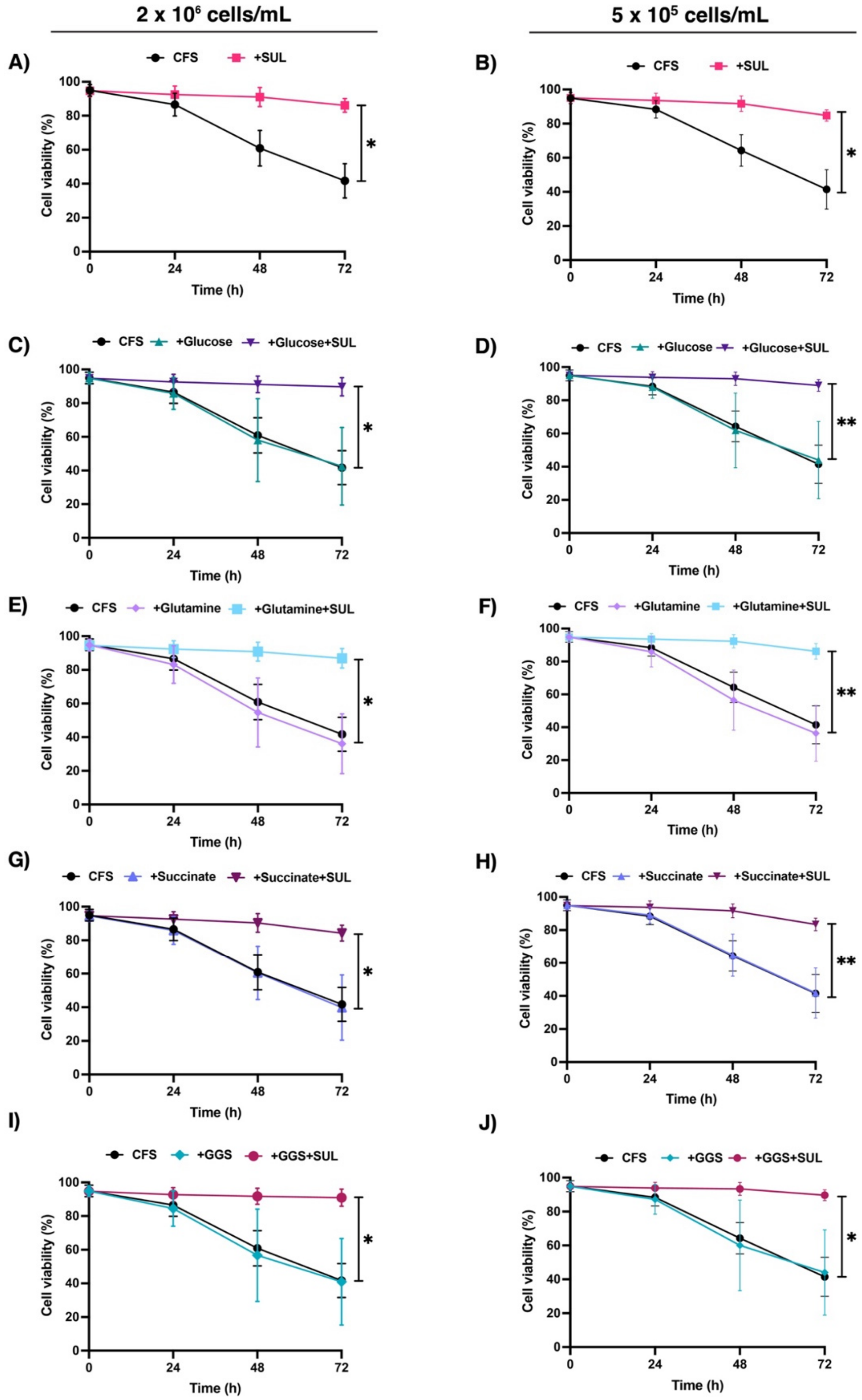

Figure S4. SUL-138 improves CAR-T cell viability during 72 h storage at 2-8 °C, regardless of mitochondrial substrate supplementation or cell concentration. Shown is the viability (7-AAD⁻) of CD19 CAR-T cell products stored for 72 h at 2-8 °C in CFS alone, or in CFS supplemented with SUL-138, glucose ± SUL-138, glutamine ± SUL-138, succinate ± SUL-138, or GGS ± SUL-138. (A, C, E, G, I) Viability at 2 × 10^6 cells/mL. (B, D, F, H, J) Viability at 5 × 10^5 cells/mL. Data are mean ± SD of n = 3 biologically independent samples. ns, not significant by one-way ANOVA with Tukey's multiple-comparison test. GGS: combination of glucose, glutamine, and succinate.
